# Artificial Intelligence in Radiology for Ethiopia

**DOI:** 10.4314/ejhs.v34i1.1S

**Published:** 2024-10

**Authors:** Kumlachew Abate Mekonen, Shimels Hussien Mohammed, Tesfaye Kebede, Alemayehu Bedane, Ashenafi Aberra Buser

**Affiliations:** 1 Department of Radiology and Medical Radiologic Technology, School of Medicine, St. Paul's Hospital Millennium Medical College, Addis Ababa, Ethiopia; 2 Department of Public Health, School of Public Health, St. Paul's Hospital Millennium Medical College, Addis Ababa, Ethiopia; 3 Department of Radiology, School of Medicine, Addis Ababa University, Addis Ababa, Ethiopia

The field of radiology is intrinsically linked to cutting-edge technologies, with the professional community demonstrating a sustained adoption of innovations (1). Radiology plays a crucial role in early diagnosis, staging, and follow-up care. Despite significant progress, challenges remain in clinical efficacy, uniform deployment, and pricing models (1-4). Artificial intelligence (AI) is emerging as a transformative force, optimizing workflow processes, including image acquisition, analysis, and reporting (5). While these advanced technologies hold promise for addressing chronic challenges in accessing quality radiological services in low-income countries like Ethiopia, their benefits are often unevenly distributed, contributing to a global AI divide that exacerbates existing health disparities (6).

Ethiopia's estimated population is around 120 million, yet the radiologist-to-population ratio is alarmingly low at 1:350,000 (7). The country faces significant disparities in the coverage and quality of healthcare services, including radiology (8). Like many low-income countries, Ethiopia grapples with a severe shortage of radiologists, limited access to basic and advanced imaging equipment, and inadequate infrastructure for delivering diagnostic imaging services. Less than 20% of healthcare facilities have ultrasound machines, about 5% are equipped with X-ray machines, and only 1% have CT scanners. Moreover, facilities often lack reliable electricity, internet connectivity, and digital data storage capabilities (7,8).

Ethiopia's unique disease burden complicates matters further; infectious diseases such as malaria, HIV/AIDS, and tuberculosis remain leading causes of morbidity and mortality, while the incidence of non-communicable diseases is rising. Timely and accurate diagnoses are essential for managing these dual burdens, underscoring the urgent need for innovative solutions (7).

Numerous AI applications are being developed in radiology that have revolutionized disease detection, management, and prognosis in high-income countries (3). AI tools can enhance the efficiency, accuracy, and accessibility of imaging services while also augmenting the capabilities of technologists and radiologists through decision support, automation of image acquisition, post- processing, and interpretation. This enables the limited pool of professionals to focus on more complex tasks. Computer-aided detection technologies assist in the early detection of diseases such as tuberculosis and breast cancer, facilitating timely interventions and improving patient outcomes (1,9,10).

To harness the benefits of AI in radiology, several challenges must be addressed. These include the establishment of secure data infrastructures, AI models tailored to local datasets, ethically developed and culturally appropriate solutions, and the effective integration of AI solutions into existing healthcare systems. Collaborative platforms involving global and local stakeholders, such as academic institutions, technology companies, and policymakers, are essential. Additionally, capacity-building and skills development are crucial to empower healthcare professionals to fully leverage AI's potential in radiology. Integrating AI education into curricula and establishing centers of excellence can help foster a strong foundation of AI-proficient radiologists and technologists (1,10).

The inclusion of AI-driven solutions in radiology holds significant promise for overcoming chronic challenges and transforming healthcare delivery. As the global medical community embraces the AI revolution, it is essential that Ethiopia keeps pace. Through collaborative efforts, strategic investments, and a commitment to innovation, the country can unlock the transformative potential of AI in radiology.
